# Factors associated with umbilical cord clamping in term newborns^*^

**DOI:** 10.1590/1980-220X-REEUSP-2021-0423

**Published:** 2022-03-28

**Authors:** Juliana Karine Rodrigues Strada, Leticia Becker Vieira, Helga Geremias Gouveia, Thais Betti, Wiliam Wegner, Cecília Drebes Pedron

**Affiliations:** 1 Universidade Federal do Rio Grande do Sul, Escola de Enfermagem, Porto Alegre, RS, Brazil.

**Keywords:** Umbilical Cord, Obstetric Nursing, Obstetrics, Records, Infant, Newborn, Neonatology, Cordón Umbilical, Enfermería Obstétrica, Obstetricia, Registros, Recién Nacido, Neonatología, Cordão Umbilical, Enfermagem Obstétrica, Obstetrícia, Registros, Recém-Nascido, Neonatologia

## Abstract

**OBJECTIVE:**

To identify factors associated with umbilical cord clamping in term newborns and to compare the recording of clamping time in the medical record with what was observed.

**METHOD:**

Cross-sectional study, with 300 mothers-infants, in a university hospital. Clamping time and medical records were observed, and a structured questionnaire was applied to postpartum women for sociodemographic variables. Bivariate analysis, multivariate Poisson Regression model, and Kappa concordance test were performed.

**RESULTS:**

The percentage of late/optimal clamping observed was 53.7%. The associated factors were skin-to-skin contact in the delivery room (PR = 0.76; 0.61–0.95; p = 0.014), position of the newborn below the vaginal canal (PR = 2.6; CI95%: 1.66–4.07; p < 0.001), position of the newborn at the vaginal level (PR = 2.03; CI95%: 1.5–2.75; p < 0.001), and need for newborn resuscitation in the delivery room (PR = 1.42; CI95%; 1.16–1.73; p = 0.001). Kappa concordance level of the professionals, records compared to the observation was: nurse 0.47, obstetrician 0.59, and pediatrician 0.86.

**CONCLUSION:**

the identification of associated factors and the comparison between recording and observing the clamping time can help in the planning and implementation of improvements for adherence to good practices at birth.

## RESUMO

**Objetivo:**

identificar fatores associados ao clampeamento do cordão umbilical em neonatos a termo e comparar o registro do tempo de clampeamento no prontuário com o observado.

**Método:**

estudo transversal, com 300 mães-bebês, em hospital universitário. Observaram-se tempo de clampeamento e registros em prontuário, sendo aplicado questionário estruturado às puérperas para variáveis sociodemográficas. Procedeu-se análise bivariada, modelo de Regressão de Poisson multivariado e teste de concordância Kappa.

**Resultados:**

o percentual de clampeamento tardio/oportuno observado foi 53,7%. Os fatores associados foram contato pele-a-pele em sala de parto (RP = 0,76; 0,61–0,95; p = 0,014), posição do recém-nascido abaixo do canal vaginal (RP = 2,6; IC95%: 1,66–4,07; p < 0,001), posição do recém-nascido no nível vaginal (RP = 2,03; IC95%: 1,5–2,75; p < 0,001) e necessidade de reanimação do recém-nascido em sala de parto (RP = 1,42; IC95%; 1,16–1,73; p = 0,001). O nível de concordância Kappa do registro dos profissionais comparado à observação foi: enfermeira 0,47, obstetra 0,59 e pediatra 0,86.

**Conclusão:**

a identificação dos fatores associados e a comparação entre registro e observação do tempo de clampeamento podem auxiliar no planejamento e implementação de melhorias para adesão às boas práticas no nascimento.

## RESUMEN

**Objetivo:**

Identificar factores asociados al clampaje del cordón umbilical en neonatos a término y comparar el registro del tiempo de clampaje en el prontuario con el observado.

**Método:**

Estudio transversal, con 300 madres-hijos, en hospital universitario. Se observó tiempo de clampaje y registros en prontuario, siendo aplicado cuestionario estructurado a las puérperas para variables sociodemográficas. Se utilizó análisis bivariado, modelo de Regresión de Poisson multivariado e índice de concordancia Kappa.

**Resultados:**

el porcentaje de clampaje tardío/oportuno observado fue 53,7%. Los factores asociados fueron contacto piel con piel en sala de partos (RP = 0,76; 0,61–0,95; p = 0,014), posición del recién nascido abajo del canal vaginal (RP = 2,6; IC95%: 1,66–4,07; p < 0,001), posición del recién nacido en el nivel vaginal (RP = 2,03; IC95%: 1,5–2,75; p < 0,001) y necesidad de reanimación del recién nacido en sala de partos (RP = 1,42; IC95%; 1,16–1,73; p = 0,001). El nivel de concordancia Kappa del registro de los profesionales comparado a la observación fue: enfermera 0,47, obstetra 0,59 y pediatra 0,86.

**Conclusión:**

la identificación de los factores asociados y la comparación entre registro y observación del tiempo de clampaje puede auxiliar en la planificación e implementación de mejorías para la adhesión a las buenas prácticas de nacimiento.

## INTRODUCTION

Adaptation to extrauterine life, despite occurring naturally, is a moment of fragility and requires special care to ensure an adequate physiological transition. It is known that the minority of newborns need maneuvers to establish breathing and circulation at birth, requiring attention to the interventions performed^(1)^.

Currently, care for newborns in the delivery room has been updated based on new evidence, with the purpose of modifying and reducing interventions that are demonstrably unnecessary and even contrary to health maintenance and adequate physiological transition^(2)^. Umbilical cord clamping is one of the procedures for the newborn that has been going through a transition process regarding the opportune moment for its performance, since it can affect the quality of the care provided^(3)^.

The World Health Organization (WHO) recommends umbilical cord clamping after complete cessation of pulsation, between one and three minutes after birth, defined as optimal or delayed clamping. When it occurs before this period, clamping is defined as early/immediate^(4)^.

Among the benefits of delayed/optimal umbilical cord clamping for the term newborn, we can mention an addition of 80 ml of placental blood, when clamping occurs 60 seconds after birth, increasing to 100 ml when it is performed up to three minutes after birth. Furthermore, it provides an iron supply of 40 to 50 mg/kg, which contributes to the reduction of iron deficiency in the first year of life^(5,6,7)^. Moreover, higher rates of exclusive or predominant breastfeeding after hospital discharge and assistance in maintaining body temperature stand out^(8)^ and better neurologic outcomes are seen in full-term newborns from birth to 4 years of age^(9)^. Despite the benefits shown, high rates of early/immediate clamping are still identified, characterizing an outdated care protocol^(5,10,11)^.

The obstacles highlighted for the occurrence of delayed/optimal umbilical cord clamping are the lack of elucidation about the appropriate moment and situation for the practice, due to the lack of consensus in the recommendations over the years^(3)^ and because early/immediate clamping was previously part of the active management of the third stage of delivery^(12)^. In addition, the need to optimize time in newborn care, and the existence of a hypotheses that delayed/optimal clamping could result in negative effects for newborns, such as polycythemia and neonatal jaundice, are evidenced as obstacles^(13)^. Thus, health workers continue to routinely perform early/immediate clamping^(5,10,11)^.

The relevance of this research is that it identified gaps in the production of knowledge in national studies and lack of clinical consensus regarding the appropriate time to perform the umbilical cord clamping. It is also important because it deals with an essential practice in the integral care of the newborn, which involves important results when performed in an optimal manner.

It is understood that data from this research may support the proposition of readjustment/updating of the care protocol aimed at the practice of clamping, contributing to the healthy development of the newborn by allowing it to receive the benefits of delayed/optimal clamping of the umbilical cord. In view of the foregoing, this study aimed to identify factors associated with umbilical cord clamping in term newborns and to compare the recording of clamping time in the medical record with what was observed.

## METHOD

### Design of Study

This is a cross-sectional, observational, and analytical study.

### Population

The population consisted of women and newborns.

### Local

The study was carried out at the obstetric center and obstetric hospitalization unit of a university hospital in southern Brazil. The Institution was chosen because it is a reference in obstetric care, mainly because it excels in the implementation of protocols and policies related to good practices in labor and birth.

The institution under study has been accredited as a Baby-Friendly Hospital since 1997, joined the *Rede Cegonha* (Maternal/Child Health Services) in 2011 and, in 2017, joined the APICE ON (Improvement and Innovation in Care and Teaching in Obstetrics and Neonatology), strategies aimed at qualifying the processes related to delivery and childbirth in institutions, with evidence-based practices.

In 2018, there were 3514 births at this institution, of which 2165 were vaginal and 1349 were C-sections, mostly attended at the Brazilian Public Health System (*SUS*).

### Sample Definition

Data collection took place from March to October 2019.

For sample calculation, a study analyzing the impact of clamping time on iron reserve of term newborns was considered, which showed 77% of early/immediate clamping^(5)^. Considering a confidence level of 95% and a margin of error of 5%, a sample size of 273 births was reached. The final sample calculated was of 300 births, adding 10% for occasional losses. The software used for sample calculation was *WINPEPI* version 11.65.

**Chart 1.**
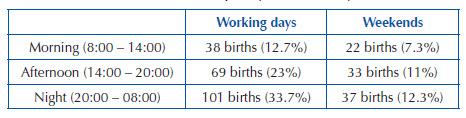
Distribution of the sample by shift and days of the week.

The distribution of participants was organized by shifts, considering the medical team working hours, working days (Monday, Tuesday, Wednesday, Thursday and Friday), and weekends (Saturday and Sunday), to ensure representativeness of each stratum. For this, the number of births per shift and per weekdays equivalent to one month (base month – September 2018) was considered, and a proportional distribution was subsequently carried out considering the sample size of the present study (Chart 1).

### Selection Criteria

Inclusion criteria were: women who had a vaginal or C-section delivery at the obstetric center of the institution being studied, admitted through the Brazilian Public Health System, with a gestational age equal to or greater than 37 weeks and who had a newborn with a birth weight equal to or greater than 2500 grams. Isoimmunized puerperal women diagnosed with HIV and HTLV, cases of death and fetal malformation, twinning, those who arrived at the institution in the expulsive period and those hospitalized through a private health provider (health plan) or at their own expense (private) were excluded.

After identifying the inclusion criteria, the women were selected for convenience, following the order of births. The maximum number of births observed per shift was three. For women who accepted to participate in the research, passive observation of umbilical cord clamping was performed at birth.

### Data Collection

In the vaginal delivery/surgery room, the practice was observed and the umbilical cord clamping time was measured using a professional stopwatch (Vollo VL510^®^).

Following birth observation, data collection, in electronic medical records, of the variables weight and birth characteristics of the newborn was complemented, to determine the inclusion of the newborn in the study. For those included in the sample, after 12 hours of birth, a structured questionnaire was applied to the puerperal woman at the Obstetric Inpatient Unit.

After the binomial was discharged, data related to the registration of the practice of clamping the umbilical cord by different professionals (nurse, obstetrician, and pediatrician) and data on the newborn’s hospitalization were collected in electronic medical records. It should be noted that, in this institution, all deliveries are attended exclusively by obstetricians.

The dependent variable was the umbilical cord clamping time, data collected through observation, measured in seconds and categorized as early/immediate umbilical cord clamping (<60 seconds) and delayed/optimal clamping of the umbilical cord (≥60 seconds). The independent variables included sociodemographic and prenatal characteristics, birth data, observations related to the practice of umbilical cord clamping, information on the practice of clamping recorded by the nurse, obstetrician and pediatrician, and data from the newborn’s hospitalization until discharge.

### Data Analysis

The database and statistical analyses were performed using the software *Statistical Package for the Social Sciences* (SPSS), version number 21. Descriptive and analytical analysis procedures were performed. Quantitative variables were evaluated by mean and standard deviation or median and interquartile range. Categorical variables were expressed as absolute and relative frequencies. The association between categorical variables was performed using the Chi-Square test, complemented by the analysis of adjusted residuals; if required, and for controlling confounding factors, the multivariate Poisson Regression model was applied to evaluate factors independently associated with early/immediate clamping. The criterion used for the insertion of the variable in the multivariate model was having a p value <0.20 in the bivariate analysis. The significance level adopted was 5% (p < 0.05).

To compare the records in electronic medical records related to the time and classification of umbilical cord clamping with data obtained through observation, Kappa concordance test was used, in which the value < 0 was considered as no concordance, 0 to 0.19 as poor concordance, 0.20 to 0.39 mild concordance, 0.40 to 0.59 moderate concordance, 0.60 to 0.79 substantive concordance, and 0.80 to 1.0, almost perfect concordance^(14)^.

### Ethical Aspects

The research project was approved in 2019, with number 3.203.072 and was prepared and conducted in accordance with the ethical precepts determined by Resolution 466/2012 of the National Health Council^(15)^.

For women who agreed to participate in the study, a Free and Informed Consent Form was applied.

## RESULTS

Of the 300 mother-infant pairs participating in this study, 53.7% underwent delayed/optimal umbilical cord clamping. The median umbilical cord clamping time was 70.5 seconds, with 25th percentile = 26 seconds and 75th percentile = 132 seconds.

Table 1 describes the maternal sociodemographic characteristics, prenatal data, and newborn characteristics. It should be highlighted that the variable showing statistical significance was maternal color/race (p = 0.004), with the predominance of white color (59.3%). The sample consisted of mothers aged between 20 and 35 years (70%) who had performed their adequate prenatal care (78.3%), with most newborns being AGA (77.7%). However, these data were not statistically significant.

**Table 1.** Sociodemographic, prenatal and newborn characteristics, Porto Alegre, Rio Grande do Sul, Brazil, 2019.

Variables	n (%)	p
**Maternal age**		**0.329**
<20 years	45 (15)	
20 to 35 years	210 (70)	
≥35 years	45 (15)	
**Self-declared color**		**0.004**
White	178 (59.3)	
Black	67 (22.3)	
Brown/Brunette/Mulatto	53 (17.7)	
Yellow	2 (0.7)	
**Years of complete study**		**0.650**
<8 years	65 (21.7)	
≥8 years	235 (78.3)	
**PN visits**		**0.502**
<8 consultations	80 (26.7)	
<8 consultations	220 (73.3)	
**Newborn sex**		**0.804**
Female	148 (49.3)	
Male	152 (50.7)	
**Newborn weight**		**0.929**
2500 to 2999	81 (27)	
3000 to 3999	195 (65)	
≥4000	24 (8)	
**Newborn classification**		**0.606**
SGA	43 (14.3)	
AGA	233 (77.7)	
LGA	24 (8)	

The data presented in Table 2 refer to data obtained through direct observation of the practice of umbilical cord clamping and birth data obtained from electronic medical records. It should be noted that the moment of clamping was defined by the pediatrician in 70.7% of the sample and that most newborns (91.7%) had 1-minute Apgar ≥7. It was observed that 35.7% of newborns required resuscitation maneuvers.

**Table 2.** Observation data of the practice of umbilical cord clamping and birth data, Porto Alegre, Rio Grande do Sul, Brazil, 2019.

Variables	n (%)	p
**Delivery**		**0.002**
Vaginal	155 (51.7)	
Vaginal with forceps	12 (4)	
C-section	133 (44.3)	
**Clamping moment**		**<0.001**
Defined by obstetrician	88 (29.3)	
Defined by the pediatrician	212 (70.7)	
**Newborn position during transfusion**		**<0.001**
Below the vaginal canal	27 (9)	
Vaginal level	34 (11.3)	
Abdominal/thoracic level	239 (79.7)	
**Newborn day and time of birth**		**0.036**
Day of week/morning	38 (12.7)	
Day of week/afternoon	69 (23)	
Day of week/night	101 (33.7)	
Weekend/morning	22 (7.3)	
Weekend/afternoon	33 (11)	
Weekend/night	37 (12.3)	
**1-minute Apgar**		**<0.001**
<7	25 (8.3)	
≥7	275 (91.7)	
**Obstetric complications at childbirth**		
Uterine hypotonia	25 (8.3)	0.638
Increased bleeding	30 (10)	0.479
Hypertensive peak	5 (1.6)	0.122
**Newborn complications until discharge**		
Jaundice	35 (11.7)	0.919
Polycythemia	3 (1)	0.300
Breathing difficulty	45 (15)	0.067
Newborn resuscitation required	107 (35.7)	<0.001
**Amniotic fluid appearance**		**0.003**
Clear amniotic fluid	238 (79.3)	
Meconium-stained amniotic fluid	62 (20.7)	
Breastfeeding in the vaginal/surgery delivery room	94 (31.3)	<0.001
Skin-to-skin contact in a vaginal/surgery delivery room	200 (66.7)	<0.001

Table 3 presents the bivariate analysis of factors associated with umbilical cord clamping. The variables that had statistical significance were type of delivery (p = 0.002), professional who defined the moment of umbilical cord clamping (p < 0.001), position of the newborn in relation to the mother at the time of placental transfusion (p < 0.001), day and time of birth of the newborn (p = 0.036), 1-minute Apgar score (p < 0.001), need for resuscitation of the newborn immediately at birth (p < 0.001), appearance of amniotic fluid (p = 0.003), breastfeeding (p < 0.001), and skin-to-skin contact in the delivery room (p < 0.001).

**Table 3.** Bivariate analysis of factors associated with umbilical cord clamping, Porto Alegre, Rio Grande do Sul, Brazil, 2019.

Variables	Early/immediate n (%)	Late/optimal n (%)	P
**Delivery**			**0.002**
Vaginal	60 (43.2)	95 (59)*	
Vaginal with forceps	3 (2.2)	9 (35.4)	
**C-section**	76 (54.7)*	57 (5.6)	
Clamping moment			**<0.001**
Defined by obstetrician	56 (40.3)*	32 (19.9)	
Defined by the pediatrician	83 (59.7)	129 (80.1)*	
**Newborn position during transfusion**			**<0.001**
Below the vaginal canal	27 (19.4)*	0 (0)	
Vaginal level	34 (24.5)*	0 (0)	
Abdominal/thoracic level	78 (56.1)	161 (100)*	
**Newborn day and time of birth**			**0.036**
Day of week/morning	18 (12.9)	20 (12.4)	
Day of week/afternoon	29 (20.9)	40 (24.8)	
Day of week/night	52 (37.4)	49 (30.4)	
Weekend/morning	16 (11.5)*	6 (3.7)	
Weekend/afternoon	11 (7.9)	22 (13.7)	
Weekend/night	13 (9.4)	24 (14.9)	
**1-minute Apgar**			**<0.001**
<7	22 (15.8)*	3 (1.9)	
≥7	117 (84.2)	158 (98.1)*	
Newborn resuscitation required	70 (50.4)*	37 (23)	<0.001
**Amniotic fluid appearance**			**0.003**
Clear amniotic fluid	100 (71.9)	138 (85.7)*	
Meconium-stained amniotic fluid	39 (28.1)*	23 (14.3)	
Breastfeeding in a vaginal delivery room/surgery	29 (20.9)	65 (40.4)*	<0.001
Skin-to-skin contact in the vaginal/surgery delivery room	76 (54.7)	124 (77)*	<0.001

*Variables with a statistically significant association.

After adjusting for confounding factors, the variables position of the newborn at the time of placental transfusion (p < 0.001), newborn resuscitation required in a vaginal/ surgery delivery room (p < 0.001), and skin-to-skin contact in the vaginal/surgery delivery room (p<0.001) remained significantly associated with the classification of umbilical cord clamping, as shown in Table 4.

**Table 4.** Multivariate analysis of factors associated with early/ immediate umbilical cord clamping, Porto Alegre, Rio Grande do Sul, Brazil, 2019.

Variables	Multivariate PR (95%CI)	P
**Newborn position during transfusion**		
Below the vaginal canal	2.60 (1.66 – 4.07)	<0.001
Vaginal level	2.03 (1.50 – 2.75)	<0.001
Abdominal/thoracic level	1.00	
**Newborn resuscitation required**		
Yes	1.42 (1.16 – 1.73)	0.001
No	1.00	
**Skin-to-skin contact in the vaginal/surgery delivery room**		
Yes	0.76 (0.61 – 0.95)	0.014
No	1.00	

Electronic medical records of different professionals, related to the time and classification of umbilical cord clamping, were compared with data obtained through observation. It was found that, according to the records made by the nurse, early/immediate clamping was performed in 45 (15%) newborns and the Kappa concordance level was 0.47 in relation to the observed practice. It should be noted that the nurse did not record the practice in 79 (26.3%) births.

Regarding the information recorded by the obstetrician, it was evidenced that the prevalence of early/immediate umbilical cord clamping was 11.7%, equivalent to 35 births, and the Kappa concordance level was 0.59 when compared to the observation of practice. There were no recordings in 132 (44%) births.

The pediatrician’s records showed that the prevalence of early/immediate umbilical cord clamping was 37% and Kappa concordance level was 0.86. There was no record of the practice in 21 (7%) births.

Despite the lack of registration of the practice of umbilical cord clamping by professional category, it should be noted that there was a record entered by at least one of the professionals in all the medical records of the study.

## DISCUSSION

This study evidenced that most newborns underwent delayed/optimal clamping of the umbilical cord. Rates similar to the present study were found in a retrospective cohort research carried out at a Baby-Friendly Hospital in Minas Gerais and in a cross-sectional study carried out in a tertiary hospital in Nepal, which showed a percentage of delayed/optimal clamping of 52%^(16,17)^. Furthermore, a study carried out in Turkey, which evaluated the use of routine interventions at birth, also showed a percentage similar to the present study, with 55.3% of delayed/optimal clamping^(10)^.

Findings on delayed/optimal clamping of the present study are similar to other studies carried out in the country and worldwide. Furthermore, they demonstrate that despite the existence of policies, protocols, and studies recommending and evidencing the beneficial effects of delayed/optimal clamping, the results may indicate that health workers are resistant to changing their practices.

Research investigating the clinical practices of umbilical cord clamping through interviews with professionals reiterate this fact, since they show that professionals, for the most part, are aware of the proper way to perform the procedure^(18,19)^. Existing knowledge, however, is not reflected in daily practice^(20)^ and in the data found in studies evaluating clinical practice, which still show low rates of delayed/optimal clamping^(10,16,17)^.

Regarding the need for resuscitation in the delivery room, a factor significantly associated with early/immediate umbilical cord clamping, it was shown that 35.7% of newborns required resuscitation maneuvers and most (65.4%) underwent early/immediate umbilical cord clamping. The need for resuscitating the newborn in the delivery room increased the chance of early/immediate clamping by 42%.

A study evaluating the factors associated with umbilical cord clamping in a large hospital in Nepal showed that the newborns who underwent any intervention following delivery were less likely to undergo delayed/optimal umbilical cord clamping when compared to those who received no intervention^(17)^. Possibly this is because when the newborn requires special support for stabilization, clamping is performed quickly for the child to be taken to the care required^(20)^. However, there are safe recommendations that it is possible to perform resuscitation maneuvers with the newborn close to the mother and with the umbilical cord still intact^(21–23)^.

A study conducted in California focused on high-risk deliveries observed the effectiveness of a mobile resuscitation platform at the mother’s bedside to keep the umbilical cord intact throughout care received. This study showed that the use of this strategy is viable, safe, and effective, requiring only logistical adjustments^(23)^.

The position of the newborn at the vaginal level also showed a statistically significant association with early/immediate umbilical cord clamping. Newborns that were kept at and below the vaginal level, that is, both below the maternal abdomen, were respectively 2.03 and 2.6 times more likely to undergo early/immediate clamping.

For a long time, it was believed that gravity influenced the amount of cord blood received by the newborn. Therefore, the recommendation was that the newborns remained at or below the vaginal level until umbilical cord clamping occurred^(24)^, a practice considered uncomfortable for the professional who is caring for the newborn and a triggering factor for the occurrence of early/immediate clamping^(25)^, which may have occurred in the present study.

It is currently known that the action of gravity does not influence the amount of umbilical cord blood provided to the newborn. A study assessing the weight difference in newborns kept in the vaginal introitus and abdominal region found a difference of 3g in weight gain, showing that mothers can hold babies during the procedure, improving bonding and obstetric adherence to the procedure^(25)^.

Skin-to-skin contact showed a statistically significant association with delayed/optimal clamping in the present study, appearing as a protective factor for delayed/optimal clamping, reducing the probability of early/immediate clamping by 24%. It is known that establishing spontaneous ventilation before performing umbilical cord clamping improves venous return and pulmonary blood flow, improving cardiac output and protecting the newborn from blood pressure fluctuations^(22)^. Thus, it is possible that the newborns subjected to optimal/delayed clamping have presented better conditions for the team to consider the effectiveness of skin-to-skin contact in the delivery room possible.

As for the records made by the different professionals responsible for umbilical cord clamping, some weaknesses were observed. Absence of the practice records or even lack of consensus regarding the classification of clamping was observed, evidenced by inconsistent records and by different records regarding classifications of the type of clamping for the same newborn. Furthermore, overestimation of the rate of delayed/optimal clamping by professionals was observed, since all of them recorded percentages that were higher than those obtained through observation. This overdimensioning, added to the lack of homogeneity of the record among professionals, can be explained by the existence of different concepts about the definition of clamping^(3)^ and the lack of applicability of the institutional guidelines recommended^(26)^, which can lead professionals to classify the procedure in different ways.

A study, which described how the change in the practice of umbilical cord clamping and its recording occurred within a health team, through the creation of institutional guidelines, reported that through protocols and teamwork it was possible to succeed in the implementation of delayed/optimal clamping as a routine practice and, also, to replicate the strategy in five other institutions^(27)^, reinforcing the importance of standardizing the procedure and raising professional awareness about the implementation of good care practice.

The identification of factors associated with umbilical cord clamping and the comparison of records with data obtained through observation can help professionals reflect on their daily practice, encourage them to keep up to date, seeking to build strategies for the implementation of effective actions in care procedures and, thus, increase the percentage of delayed/optimal clamping.

A limitation of the study to be recognized is the use of the convenience sampling technique, which is subject to a potential risk of selection bias. Another limitation is related to the fact that it was carried out in a single institution, which may limit the generalization of data.

## CONCLUSION

This study evidenced that most newborns underwent delayed/optimal umbilical cord clamping. The factors showing a statistically significant association in the present study were the need for resuscitation of the newborn in the delivery room, the position of the newborn at the time of transfusion, and skin-to-skin contact in the delivery room, with the latter being a protective factor for delayed/optimal umbilical cord clamping.

In the comparison analysis of the record of the practice of umbilical cord clamping with the data obtained through observation, the professional who obtained the greatest concordance with what was observed in practice was the pediatrician. The results obtained indicated weaknesses in the records and lack of homogeneity in the information recorded by different professionals about the same patient, which indicates that, possibly, the lack of consensus in the literature regarding the appropriate time and situations for performing umbilical cord clamping have reflected in the practice and in the recording by professionals of this institution.

The review of institutional protocols on the practice together with managers and a multidisciplinary team is suggested to standardize recommendations on clamping, aiming at adjusting routines to good newborn care practices, based on scientific evidence. The need to qualify the records and make professionals aware of the importance of complete and homogeneous records is highlighted, to avoid interference in the quality of care.

## ASSOCIATE EDITOR

Ivone Evangelista Cabral
